# Kynurenine pathway metabolites as potential biomarkers in age-related macular degeneration: an ELISA-based prospective study

**DOI:** 10.1186/s40942-025-00757-3

**Published:** 2025-11-28

**Authors:** Ahmet Dundar, Songul Cetik Yildiz

**Affiliations:** Health Services Vocational School, Department of Medical Services and Techniques, Mardin Artuklu University, Mardin, Turkey

**Keywords:** Age-related macular degeneration (AMD), Kynurenine pathway, Tryptophan metabolism, Biomarkers, ROC analysis

## Abstract

**Objectives:**

Age-related macular degeneration (AMD), in which oxidative stress, inflammation and metabolic imbalances play a role in its pathogenesis, is one of the leading causes of irreversible vision loss. The kynurenine (KYN) pathway, one of the principal routes of tryptophan (TRP) metabolism, constitutes an important mechanism in retinal neurodegeneration. Based on this information, our study aimed to compare the serum TRP, KYN, kynurenic acid (KYNA), 3-hydroxykynurenine (3HK), 3-hydroxyanthranilic acid (3HAA) and, quinolinic acid (QA) levels of AMD patients and to investigate the diagnostic values ​​of these biomarkers.

**Methods:**

Serum samples were collected from AMD patients and control groups. TRP, KYN, KYNA, 3HK, 3HAA, and QA levels were measured using a commercial ELISA method. KYN pathway activity, KYN/TRP and, KYNA/3HK ratios were also assessed. Mann-Whitney U test, ROC analysis, Spearman correlation were applied for statistical comparisons.

**Results:**

According to our results, 3HK was significantly higher in the AMD group, while TRP, KYN, QA, and KYNA/3HK ratio were higher in the control. ROC analysis revealed 3HK to be the strongest discriminatory marker. The KYNA/3HK ratio also provided significant diagnostic value. Correlation analysis revealed strong negative correlations between 3HK and KYN, QA, and especially KYNA/3HK. Conversely, strong positive correlations were found between KYN and KYNA/3HK, and between TRP, KYN, QA, and KYNA.

**Conclusion:**

KYN pathway metabolites exhibit significant alterations in patients with AMD. 3HK levels and the reduction of the KYNA/3HK ratio suggest a disruption of the neurotoxic–neuroprotective balance and imply that KYN pathway dysfunction may play a role in the pathogenesis of AMD. Among the biomarkers examined, 3HK displayed the highest diagnostic performance, while the KYNA/3HK ratio emerged as an additional biological indicator. These findings indicate that 3HK and the KYNA/3HK ratio may serve as potential biomarker candidates for the early diagnosis and monitoring of AMD.

## Introduction

Age-related macular degeneration (AMD) is a complex disease affecting the macula region of the eye, causing vision loss especially in individuals over the age of 50, in which the retinal pigment epithelium (RPE), photoreceptor cells, Bruch’s membrane and choroidal vascular structures are damaged by degenerative and inflammatory changes [1]. Oxidative stress, inflammation, genetic predisposition, lipid metabolism disorders, environmental factors and complement system disorders come to the fore in the pathogenesis of the disease [2]. Tryptophan (TRP) is a proteogenic amino acid that is primarily catabolized through the kynurenine (KYN) pathway in the body and also participates in other biosynthetic routes such as the production of serotonin, melatonin, and indole derivatives [3]. TRP is metabolized into KYN via indoleamine 2,3-dioxygenase (IDO) or hepatic tryptophan 2,3-dioxygenase (TDO). KYN is further processed into neuroprotective metabolites, such as kynurenic acid (KYNA), or neurotoxic metabolites, including 3-hydroxykynurenine (3HK) and quinolinic acid (QA). Other intermediates, including 3-hydroxyanthranilic acid (3HAA), also participate in this pathway, highlighting its complexity and relevance to neurophysiological processes [3, 4]. When this pathway functions properly, metabolites such as KYNA can suppress ROS formation, contribute to the prevention of synaptic excitotoxicity as NMDA receptor antagonists, and exhibit antioxidant and anti-inflammatory effects [5]. However, when the pathway shifts to toxic branches (such as 3HK, QA), oxidative stress increases, NMDA receptor stimulation and cellular damage occur, and neurodegenerative processes may accelerate [6, 7]. The KYN/TRP ratio is often used as an index of tryptophan degradation. This ratio indicates IDO/TDO activity and indicates the rate and extent of KYN formation from TRP. Increased KYN/TRP is frequently observed in inflammation or stress. This shows a potential increase in the production of toxic metabolites in nervous tissues [3, 5]. The KYNA/3HK ratio balances the neuroprotective (KYNA) and neurotoxic (3HK) branches. High KYNA and low 3HK levels denote a higher ratio, indicating a more protective profile. Conversely, low KYNA/3HK levels show a predisposition to toxic and oxidative stress. Therefore, this ratio may be a good candidate biomarker for disease activity, progression, or treatment response in retinal or systemic biomarkers. Because oxidative stress, inflammation, and cell death play a central role in the pathogenesis of AMD, the KYN pathway is directly involved in these mechanisms [1]. Prospective comparative studies on serum levels, particularly of TRP-KYN metabolites (TRP, KYN, KYNA, 3-HK, 3-HAA, QA), and their ratios in human AMD patients are scarce. Therefore, our study aims to contribute to a better understanding of the role of the KYN pathway in AMD pathophysiology by measuring these metabolite profiles and ratios in both AMD patients and control groups.

## Materials and methods

### Study design and participants

This prospective cross-sectional study was conducted at the Ophthalmology Clinic of Mardin Training and Research Hospital. Ethical approval was obtained prior to the study, and signed informed consent forms were obtained from all participants. The study cohort comprised 30 patients diagnosed with AMD disease (Group I) by a specialist ophthalmologist following a detailed ophthalmic examination and, 30 healthy individuals without an AMD diagnosis (Control = Group II).

### Diagnosis

Patients whose diagnoses were confirmed by an ophthalmologist using detailed fundus examination, optical coherence tomography (OCT), and fundus fluorescein angiography (FFA) were included in the study. Patients with drusen, hypo- or hyperpigmented areas in the macula but without choroidal neovascularization (CNV) findings (subretinal fluid formation, macular edema, serous, fibrovascular, or hemorrhagic pigment epithelial detachment, or leakage from CNV) on OCT and FFA were included in the study as dry type AMD [8].

#### Inclusion criteria

Patients aged 18 years or older who had been diagnosed with dry type AMD by an ophthalmologist were eligible to participate. Although AMD primarily affects individuals over 50 years, the lower age limit was set at 18 years to comply with ethical standards.

#### Exclusion criteria

Patients were identified as having comorbid systemic conditions such as chronic infection, diabetes, obesity (BMI > 25), glaucoma, chronic lung disease, other retinal disorders, or taking steroid medications. The control group consisted of healthy individuals who did not meet these exclusion criteria and were matched to the patient group for age and gender.

### Biochemical analyses

Blood samples were taken from healthy individuals and patients into a biochemistry tube without an anticoagulant. After coagulation, the blood samples were centrifuged at 2500 rpm for 10 min. After the serum samples were obtained in this process, the serum samples were transferred to eppendorf tubes and stored at -80 °C until the day of the study. Serum tryptophan (TRP Cat. No: SLD2253Hu), kynurenine (KYN Cat. No: SL2283Hu), kynurenic acid (KYNA Cat. No: SLD4116Hu), 3-hydroxyanthranilic acid (3HAA Cat. No: SLD4575Hu), 3-hydroxykynurenine (3HK Cat. No: SL4241Hu) and quinolinic acid (QA Cat. No: SLD4117Hu) were measured by Sandwich enzyme-linked immunosorbent assay (ELISA) method [9–12]. Furthermore, to assess the activity of the KYN pathway, the KYN/TRP ratio, a surrogate marker of IDO activity (noting that in serum TDO activity can also influence this ratio), and the KYNA/3HK ratio (representing the neuroprotective/neurotoxic balance) were calculated based on the obtained data. This method is based on the antigen-antibody interaction, and in this method, antibodies are immobilized on the solid phase of the wells of the microtiter plate. The sample containing the antigen is then added. In this way, the antigen-antibody complex is formed and the substance to be sought is determined. In the study, commercial ELISA kits from Sunlong (Sunlong Biotech Co., LTD, China) were used. The study was conducted according to the protocol of the manufacturer’s company. Absorbance values were measured at 450 nm in a BioTek (ELx800TM; Instruments, USA) ELISA reader.

### Statistical analysis

Statistical analysis of the data was performed using SPSS Version 27.0. Descriptive statistics are expressed as mean ± standard deviation (Mean ± SD) for continuous variables, and as number (n) and percentage (%). The conformity of continuous variables to a normal distribution was assessed using the Shapiro-Wilk test. The t-test was used for comparison of the normally distributed age variable between groups. The Mann-Whitney U test, a non-parametric test, was used for comparison of all other biomarkers that did not meet the normal distribution assumption. Receiver Operating Characteristic (ROC) analysis was performed to evaluate the diagnostic performance and discriminatory power of variables found to be statistically significant between groups. The Area Under the Curve (AUC) value was calculated together with the 95% Confidence Interval (CI). The AUC value was used as a measure of how well a variable discriminated between two groups. The optimal cut-off point for the biomarker with the highest diagnostic performance was determined using Youden’s J Index (Sensitivity + Specifity − 1) to determine the point that best balances sensitivity and specificity. Spearman’s Rank Correlation Coefficient (rho) was used to examine the direction and strength of the relationship between variables due to the non-normal distribution of the data. In all statistical analyses, *p* < 0.05 was accepted as the significance level.

## Results

### Demographic data

The demographic characteristics of the groups included in the study are presented in Table 1. As a result of the analyses, no statistically significant difference was found between the groups in terms of mean age (*p* = 0.716) and gender distribution (*p* = 0.795) (*p* > 0.05 for both). In the patient group, 17 participants (56.7%) were female and 13 (43.3%) were male, whereas in the control group, 16 participants (53.3%) were female and 14 (46.7%) were male.

**Table 1:** Comparison of demographic characteristics of groups

Variables	Patient group (*N* = 30)	Control group (*N* = 30)	*p*-value
Age, year (Mean ± SD)	62.47 ± 3.95	62.83 ± 3.82	0,716 ᵃ
Gender, n (%)			0,795 ᵇ
Female	17 (56.7%)	16 (53.3%)	*p* > 0.05
Male	13 (43.3%)	14 (46.7%)	*p* > 0.05

SD: Standard Deviation, ^a^ Independent Samples T-Test, ^b^ Pearson Chi-Square Test

### Comparison of biomarker levels of groups

When biomarker levels were compared between the patient and control groups, statistically significant differences were found in all variables. Median 3HK levels were significantly higher in the patient group than in the control (*p* < 0.001). However, KYN, QA, TRP, and KYNA/3HK levels were found to be statistically significantly higher in the control group (Table 2).

**Table 2:** Comparison of biomarker levels by groups (Mann-Whitney U Test)

Parameters	Patient group Median (IQR)	Control Median (IQR)	*p*-value
3HK	13.92 (1.62)	12.55 (4.60)	**< 0.001**
KYNA/3HK	4.08 (3.43)	6.39 (5.73)	**0.001**
TRP	6.64 (2.84)	8.78 (4.07)	**0.006**
KYN	56.07 (31.61)	81.60 (45.95)	**0.006**
QA	229.99 (61.50)	260.37 (34.58)	**0.026**

IQR: Interquartile Range

### Diagnostic performance of biomarkers and optimal cut-off point

The diagnostic performance of all variables found to be significant between groups was evaluated using ROC analysis. 3HK demonstrated the highest discriminatory power (The Area Under the Curve [AUC] = 0.815). The performance of all significant biomarkers in distinguishing between patient and control (ranked from highest to lowest according to AUC values) is shown (Table 3).

**Table 3:** ROC analysis results of biomarkers

Parameters	AUC	95% Confidence Interval	*p*-value
3HK	0.815	0.708–0.922	< 0.001
KYNA / 3HK	0.741	0.612–0.870	0.001
TRP	0.708	0.576–0.840	0.006
KYN	0.706	0.564–0.847	0.006
QA	0.667	0.517–0.817	0.026

**Fig. 1:**
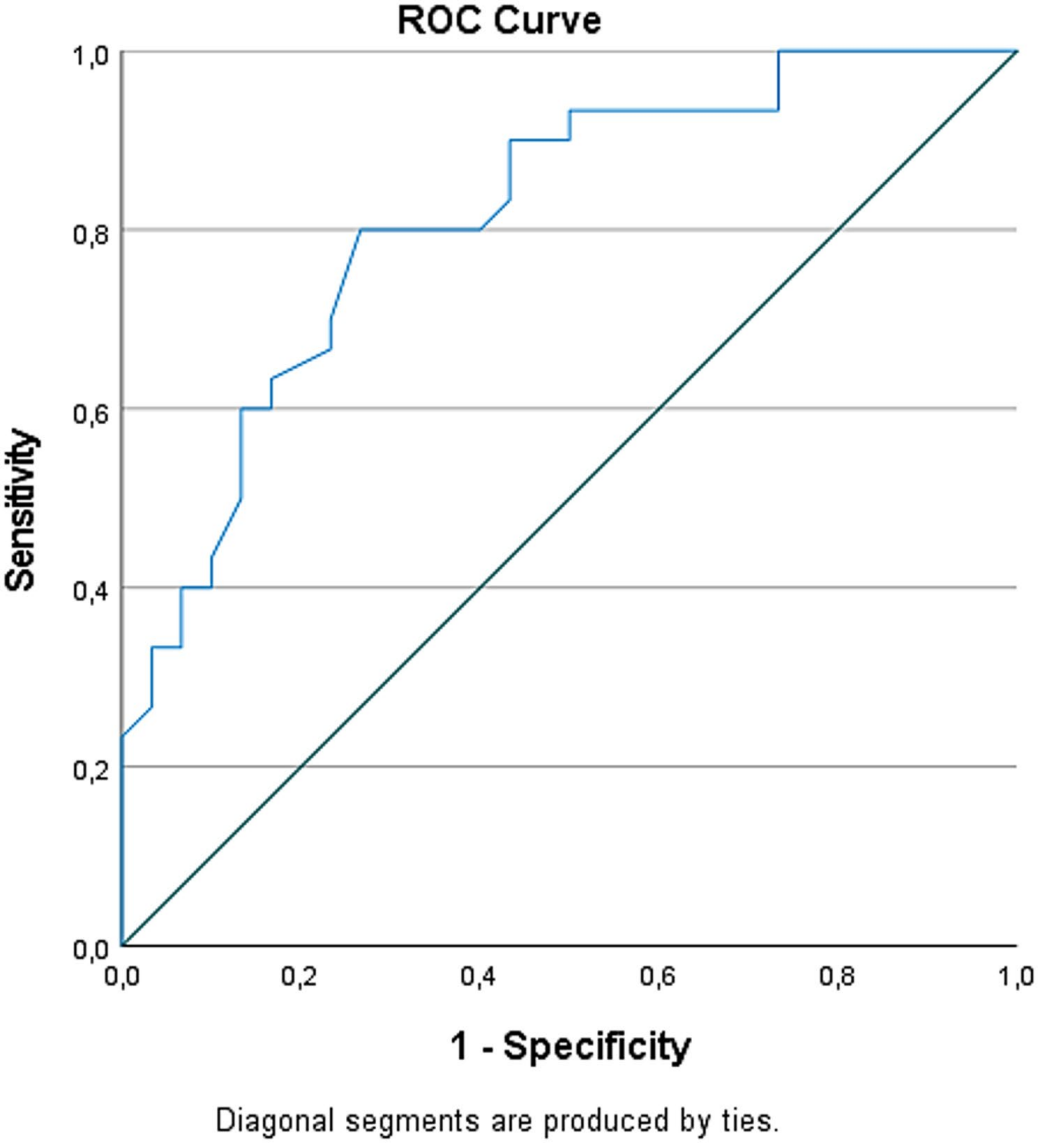
ROC curve showing the performance of 3HK in distinguishing between AMD patient and control groups. The AUC was calculated as 0.815 (95% Confidence Interval [0.708–0.922], *p* < 0.001)

The optimal cutoff point for the 3HK, which had the highest performance, was determined to be 13.5242. This cutoff value was found to best separate the groups, with 80% sensitivity and 73.3% specificity. This finding means that if the 3HK were used as a diagnostic test, this cutoff value would correctly classify 80 out of every 100 patients with disease and approximately 73 out of every 100 healthy individuals (Fig. 1).

### Correlations between variables

The relationships between all numerical variables measured in the study were examined using Spearman correlation analysis, and the results are presented in Table 4. The analysis revealed numerous statistically significant relationships, both positive and negative, among the variables. 3HK, the strongest diagnostic biomarker in the study, exhibited significant and negative correlations with many variables. The strongest of these inverse relationships was observed with the KYNA/3HK ratio (rho = -0.673, *p* < 0.01), followed by KYN (rho = -0.511, *p* < 0.01), TRP (rho = -0.497, *p* < 0.01), and QA (rho = -0.346, *p* < 0.01). Conversely, a pattern of widespread positive relationships was found among the other variables. The most striking finding in this context is the almost perfect positive correlation observed between KYN and the KYNA/3HK ratio (rho = 0.964, *p* < 0.01). Furthermore, KYN, KYNA, QA, and TRP variables were also found to exhibit consistently moderate to strong positive correlations among themselves. For example, KYN had significant positive relationships with QA (rho = 0.598, *p* < 0.01), TRP (rho = 0.462, *p* < 0.01), and KYNA (rho = 0.360, *p* < 0.01). 3HAA also exhibited weak to moderate positive relationships with this group (rho = 0.298, *p* < 0.05 with KYNA; rho = 0.296, *p* < 0.05 with QA; rho = 0.283, *p* < 0.05 with KYN). Finally, it was found that the demographic factor age did not show a statistically significant correlation with any of the biomarkers examined.

**Table 4:** Spearman correlation matrix between variables

Variables	AGE	3HK	3HAA	KYN	KYNA	QA	TRP	KYN / TRP	KYNA / 3HK
AGE	1,000	-0,202	-0,075	0,149	-0,122	-0,002	-0,026	0,159	0,175
3HK	-0,202	1,000	0,064	-0,511^**^	-0,128	-,346^**^	-0,497^**^	0,002	-0,673^**^
3HAA	-0,075	0,064	1,000	0,283^*^	0,298^*^	0,296^*^	0,047	0,261^*^	0,246
KYN	0,149	-0,511^**^	0,283^*^	1,000	0,360^**^	0,598^**^	,462^**^	0,581^**^	0,964^**^
KYNA	-0,122	-0,128	0,298^*^	0,360^**^	1,000	0,236	0,330^*^	0,150	0,379^**^
QA	-0,002	-0,346^**^	0,296^*^	0,598^**^	0,236	1,000	0,331^**^	0,246	0,564^**^
TRP	-0,026	-,497^**^	0,047	0,462^**^	0,330^*^	0,331^**^	1,000	-0,357^**^	0,520^**^
KYN / TRP	0,159	0,002	0,261^*^	0,581^**^	0,150	0,246	-0,357^**^	1,000	0,503^**^
KYNA / 3HK	0,175	-,673^**^	0,246	0,964^**^	0,379^**^	,564^**^	0,520^**^	0,503^**^	1,000

* *p* < 0.05 significant correlation level ** *p* < 0.01 significant correlation level

## Discussion

In our study, no statistically significant differences were found between the patient and control groups in terms of mean age and gender distribution. Age and gender are the most frequently controlled demographic variables for AMD and biomarker levels. Indeed, according to the findings of our study, the mean age in both groups was >62. A study similar to our findings also reported that advanced age is a major risk factor in the pathogenesis of AMD [13]. Our findings regarding gender distribution are also consistent with some studies in the literature, which have reported no significant gender differences in AMD prevalence [14, 15]. And, we acknowledge that TRP metabolism can differ between sexes, as reported by Pais et al. [16]. Although our study did not reveal a significant difference in AMD prevalence between genders, previous epidemiological studies have reported that women tend to be affected more frequently than men [17]. This gender disparity may be related to hormonal and metabolic differences that influence tryptophan metabolism and retinal vulnerability.

According to serum level comparisons, statistically significant differences were found between the AMD patient group and the healthy control group in all measured KYN pathway components. The median 3HK was significantly higher in the patient group, while KYN, QA, TRP, and the KYNA/3HK ratio were significantly higher in the control group. Studies have shown that 3HK, despite having dual (both oxidant and antioxidant) properties, is a metabolite strongly associated with oxidative stress and cell death in the retina and neural tissue. Increased 3HK levels have been shown to increase retinal damage [18, 19]. Elevated 3-HK levels have been shown to affect specific cell types, including astrocytes and neurons, leading to cellular stress and nicotinamide adenine dinucleotide (NAD+) depletion, which is considered a central mechanism of cytotoxicity [20]. In this context, the significantly elevated levels of systemic 3HK in AMD patients support that kynurenine pathway–related oxidative mechanisms may contribute to AMD pathology, although these effects are likely systemic and not limited to the retina. Furthermore, elevated 3HK levels have been reported to support a link with degenerative processes in neurodegenerative diseases such as Alzheimer’s disease [21, 22]. This points to an overlap with AMD, which is also an age-related degenerative process. There is research showing that elevated 3HK increases retinal sensitivity in retinal damage models, while KYNA supplementation is protective (preclinical study indicate that 3HK supplementation increases damage and protects against KYNA) [19]. This study supports our finding of an increase in 3HK. However, because there are few human studies specifically on AMD and serum KYN pathway metabolites, direct comparisons are limited, and therefore, our study will contribute to this field with clinical human data. Our study demonstrates significantly elevated serum 3HK levels and a decreased KYNA/3HK ratio in AMD patients, suggesting a possible role for the KYN pathway in retinal degeneration. These results are consistent with preclinical data, suggesting that KYN pathway metabolites have potential as both a biomarker and therapeutic target.

Biomarker levels were compared between AMD and control, and discriminatory power was assessed using ROC analysis. The findings indicate that 3HK levels, in particular, have high sensitivity and specificity in distinguishing the AMD group from the control. The calculated AUC value for 3HK was 0.815 (95% CI: 0.708–0.922, *p* < 0.001), demonstrating the strong diagnostic performance of this biomarker. Studies [23, 24] have reported that 3HK is associated with neurodegenerative processes and inflammatory responses, so it may be expected to play a role in the pathophysiology of AMD. The KYNA/3HK ratio was also found to be significant in distinguishing between AMD and control (AUC = 0.741, *p* = 0.001). This ratio provides information about the balance of the KYN pathway and supports that metabolic balance is disrupted in the development of AMD. Previous studies have also emphasized that KYNA has neuroprotective effects and 3HK has neurotoxic effects, and that this balance is critical for retinal health [18, 25]. TRP, KYN, and QA levels were found to differ significantly in the AMD group and had moderate discriminatory power in ROC analysis (TRP AUC = 0.708; KYN AUC = 0.706; QA AUC = 0.667). These results indicate that TRP metabolism is affected in multiple ways in AMD. Similarly, a previous study has reported that impaired TRP metabolism is associated with retinal inflammation and oxidative stress [26]. It should be noted that while the KYN/TRP ratio is commonly used as a surrogate marker of IDO activity, in serum samples its value can also be substantially influenced by TDO activity, which is primarily expressed in the liver. Therefore, interpretations of serum-based KYN/TRP ratios should consider contributions from both IDO and TDO activities, highlighting the importance of integrating other pathway metabolites for a comprehensive assessment of kynurenine pathway flux in AMD patients [27]. Given that the KYN pathway represents the main route for de novo NAD⁺ biosynthesis, its activation in AMD may also reflect a compensatory response to reduced NAD⁺ availability under oxidative or inflammatory stress conditions. This mechanism could contribute to maintaining cellular redox balance, although chronic activation might lead to an accumulation of neurotoxic intermediates such as 3HK. Our study findings suggest that various metabolites of the KYN pathway can be used as biomarkers of AMD. In particular, 3HK and the KYNA/3HK ratio stand out as potential candidate markers for early diagnosis or monitoring progression of AMD.

Relationships between biomarkers measured in the AMD group were evaluated using Spearman correlation analysis, and the data indicate that the KYN pathway, in particular, plays a critical role in AMD pathophysiology. The most striking finding in the study was that 3HK levels exhibited statistically significant and mostly negative correlations with other biomarkers. The strongest inverse correlation was found with the KYNA/3HK ratio (rho = -0.673, *p* < 0.01). This result supports the notion that the neuroprotective effects of KYNA and the neurotoxic effects of 3HK have opposing functions, as reported in the literature [18, 23], and that their biological balance is disrupted in AMD. The negative correlation between 3HK and KYN (rho=-0.511) and TRP (rho=-0.497) shows an imbalance in pathway flux and increased substrate consumption. These findings are consistent with a previous study reporting that TRP metabolism is associated with retinal inflammation [24]. In our study, the negative correlation between 3HK and QA (rho = -0.346) is also significant for AMD. Although the neurotoxic properties of QA are known [25], the inverse relationship observed here supports that different regulatory mechanisms within the pathway may play a role in the pathophysiology of AMD. A study reported that QA levels were particularly elevated in advanced neurodegenerative diseases but were more variable in retinal pathologies [28]. On the other hand, the very strong positive correlation (rho = 0.964) between KYN and the KYNA/3HK ratio is noteworthy. This finding suggests that intermediates within the pathway are significantly co-regulated and that KYN accumulation plays a critical role in KYNA production. Furthermore, moderate to strong positive correlations were found between KYN, QA, TRP, and KYNA. This suggests that TRP metabolism is holistically activated in AMD. Similar positive correlations have been reported in Alzheimer’s and Parkinson’s disease [22], supporting that neurodegenerative diseases may share common biochemical pathways. The weaker correlations between 3HAA and other metabolites suggest that this metabolite may play a secondary or supporting role in AMD. Indeed, some studies have implicated 3HAA as a secondary player in oxidative stress processes [29]. Finally, the lack of a significant correlation between age and biomarkers shows that these metabolic changes are related to the disease’s unique biochemical pathways rather than the age-related nature of AMD. Overall, these correlation analyses demonstrate that the balance between different metabolites of the KYN pathway is critical in AMD, and that 3HK and the KYNA/3HK ratio, in particular, may stand out as disease biomarkers.

The KYN pathway is the major route for de novo synthesis of NAD⁺, a crucial cofactor in cellular energy metabolism and redox homeostasis. Dysregulation of this pathway can lead to reduced NAD⁺ availability, contributing to impaired mitochondrial function and increased oxidative stress in retinal cells. Elevated levels of neurotoxic metabolites such as 3-HK may exacerbate this process by promoting NAD⁺ depletion and cellular damage [20]. Therefore, NAD⁺ imbalance may represent a key link between KYN pathway activation and the degenerative changes observed in AMD. Beyond AMD, a recent study [30] has demonstrated that dysregulation of the KYN pathway is also associated with other ocular diseases, including cataract, diabetic retinopathy, glaucoma, and pseudoexfoliation syndrome. This supports the notion that alterations in TRP metabolism may play a broader role in ocular pathophysiology.

## Conclusion

The significant differences detected in serum levels in our study, particularly the elevation of 3HK and the decrease in the KYNA/3HK ratio, can be interpreted as biochemical reflections of oxidative stress and neuroinflammatory mechanisms in retinal degeneration. ROC analyses revealed the high diagnostic performance of 3HK, while the KYNA/3HK ratio emerged as a complementary marker indicating metabolic imbalance. Correlation analyses demonstrated that the balance between KYN pathway metabolites is central to the development of AMD, and the interrelationships between these metabolites pointed to biochemical mechanisms overlapping with neurodegenerative processes. In conclusion, this study contributes to both the development of clinical diagnostic capabilities and the identification of new therapeutic targets by revealing the biomarker profile of AMD. 3HK and the KYNA/3HK ratio, in particular, can be considered strong candidate biomarkers for the early diagnosis and progression monitoring of AMD. Our findings suggest that KYN pathway metabolites are important tools not only for understanding pathophysiological processes but also as biomarkers and potential therapeutic targets that can be carried into clinical practice.

## Data Availability

No supplementary or multimedia data has been added. The data that support the findings of this study are available from the corresponding author upon reasonable request.
